# Infralimbic BDNF signaling is necessary for the beneficial effects of extinction on set shifting in stressed rats

**DOI:** 10.1038/s41386-021-01171-7

**Published:** 2021-09-08

**Authors:** Denisse Paredes, Anna R. Knippenberg, David A. Morilak

**Affiliations:** 1 Department of Pharmacology and Center for Biomedical Neuroscience, University of Texas Health Science Center at San Antonio, San Antonio, TX USA; 2 South Texas Veterans Health Care System, San Antonio, TX USA

**Keywords:** Stress and resilience, Neurotrophic factors

## Abstract

Current pharmacotherapies for posttraumatic stress disorder (PTSD) and major depressive disorder (MDD) are ineffective for many patients, and often do not restore cognitive dysfunction associated with these disorders. Behavioral therapies, such as exposure therapy, can be effective for treatment-resistant patients. The mechanisms underlying exposure therapy are not well-understood. Fear extinction as an intervention after chronic stress can model the beneficial effects of exposure therapy in rats. Extinction requires neuronal activity and protein synthesis in the infralimbic (IL) cortex for its beneficial effects. We hypothesized that extinction requires Brain-Derived Neurotrophic Factor (BDNF) activity in the IL cortex to reverse stress-induced cognitive flexibility impairments. Extinction learning reversed set-shifting deficits induced by Chronic Unpredictable Stress (CUS), tested 24 h after extinction. Blocking BDNF signaling in the IL cortex during extinction by local administration of a neutralizing antibody prevented the beneficial effects of extinction on set shifting after stress. Extinction induced activation of the BDNF TrkB receptor, and signaling pathways associated with BDNF (Akt and Erk). Administration of exogenous BDNF into IL cortex in the absence of extinction was sufficient to reverse the effects of stress on set shifting. The effects of extinction were prevented by blocking either Erk or Akt signaling in the IL cortex, whereas the effects of exogenous BDNF were dependent on Erk, but not Akt, signaling. Our observations suggest that BDNF-Erk signaling induced by extinction underlies plastic changes that can reverse or counteract the effects of chronic stress in the IL cortex.

## Introduction

Psychiatric disorders such as posttraumatic stress disorder (PTSD) and major depressive disorder (MDD), are often comorbid, and affect millions of people every year [1, 2]. These disorders share pathological features, such as maladaptive shifts in coping behavior, and deficits in executive function. Selective serotonin reuptake inhibitors (SSRIs) are the most commonly prescribed pharmacotherapy for such disorders, but only approximately 60% of patients respond to treatment. Behavioral therapies, such as exposure therapy, can be effective [3]. However, only a small percentage of patients achieve full remission after exposure therapy [4]. Deciphering the neurobiological mechanisms underlying exposure therapy could lead to the development of strategies to improve therapeutic outcomes.

Cognitive flexibility is the ability to modify previously established associations, thoughts, or responses based on changes in the environment [30]. Cognitive flexibility deficits represent a symptom dimension shared across several psychiatric disorders, are associated with increased symptom severity in PTSD [5], and are a risk factor for developing MDD [6]. Set shifting is a form of cognitive flexibility that relies on the function of the medial prefrontal cortex (mPFC) [7], a brain region susceptible to the detrimental effects of stress [8]. Set shifting is a change in response strategy (i.e., a “cognitive set”) that requires one to redirect attention from one perceptual dimension to another dimension previously irrelevant to the strategy [7]. Behavioral therapies, such as exposure therapy, address underlying cognitive dysfunction instead of targeting individual symptoms that may result from such dysfunction [9]. Exposure therapy has been shown to improve set shifting [10]. Behavioral therapies engage areas of the brain, such as the hippocampus, prefrontal cortex, and amygdala, that are affected by chronic stress [11].

Exposure therapy involves modifying one’s cognitive appraisal of fear by utilizing fear extinction (FE) learning, a form of safety learning that consists of forming a new association between a conditioned stimulus and conditioned response [12, 13]. FE in rodents mimics some of the beneficial effects of exposure therapy [14, 15]. During FE, rodents learn that a previously conditioned stimulus (e.g., a tone) no longer signals an aversive stimulus (e.g., footshock). The resulting safety memory is formed through activity in the infralimibic (IL) portion of mPFC [16]. We have shown that extinction is effective in reversing stress-induced deficits in set shifting [14]. Extinction restores responsivity of the mPFC that is compromised by chronic stress and promotes adaptive active coping behavior, similar to the effects of exposure therapy in humans [17, 18]. These effects of extinction require activity-dependent protein translation in the IL [17]. However, the molecular mechanisms that modulate IL plasticity during extinction are not known.

Brain-Derived Neurotrophic Factor (BDNF) is necessary for extinction learning and consolidation in rodents and humans alike [19, 20]. Additionally, exogenous administration of BDNF into the IL in the absence of extinction reduces fear (i.e., freezing) to the conditioned stimulus, mimicking extinction [21]. Therefore, we hypothesized that BDNF signaling in the IL may also be involved in the beneficial effects of extinction on set shifting after stress. BDNF can initiate plasticity through phosphorylation of its receptor, Tropomyosin receptor kinase B (TrkB), at tyrosine residues Y515 and Y816. Phosphorylation of Y515 activates the mitogen-activated protein kinase (MAPK)/extracellular regulated protein kinase (Erk) and phosphatidylinositol 3-kinase (PI3k)/protein kinase B (Akt) signaling pathways, both of which modulate neuronal plasticity and protein translation, and have been associated with extinction memory consolidation [22, 23], whereas TrkB phosphorylation at Y816 activates phospholipase C (PLC) signaling [24]. Dysregulation of both Erk and Akt signaling has been implicated in psychiatric disorders [25]. Erk and Akt are reduced in the mPFC and hippocampus of depressed and suicidal individuals, and studies report increases in Erk after antidepressant treatment [25]. Additionally, glucocorticoid exposure decreases phosphorylation of Erk in the hippocampus and increases immobility on the forced swim test [26]. We hypothesized that BDNF activity in the IL during extinction may be necessary to induce functional changes in stressed animals as a result of learning-induced activation of MAPK/Erk and/or PI3k/Akt signaling. Portions of this work have been presented in abstract form [27].

## Materials & methods

### Animals

A total of 258 male and naturally cycling female rats (Envigo, 225–249 g) were group-housed on a 12/12 hr light cycle (lights on at 07:00 hr), with same-sex cagemates upon arrival. After 1 week of acclimation, they were single-housed and provided with food and water *ad libitum*. Experiments took place between 08:00-17:00 hr, during the lights-on portion of the cycle. All procedures were in accordance with NIH guidelines and approved by the UTHSA Institutional Animal Care and Use Committee.

### Fear conditioning and extinction

Two days prior to the start of Chronic Unpredictable Stress (CUS) procedures, rats were habituated to two contexts in sound-attenuating cabinets for 15 min each. Context A consisted of a metal conditioning chamber (30.5 × 25.4 × 30.5 cm; model H10-11RC-TC-Sf) with square walls and a grid floor attached to a shock box (model H13–15). Context B was not associated with a shock and was in a different chamber, with pink and white vinyl floor and white vinyl circular walls.

### Day 0 - Fear conditioning

Rats received fear conditioning or tone control treatment in Context A the day before the start of CUS. Fear conditioning consisted of four pairings of a tone (10 kHz, 75 dB, 20 s) coterminus with a footshock (0.8 mA, 0.5 s), with an inter-trial interval of 120 s. Freezing during each tone was quantified videographically by FreezeView software (ActiMetrics #ACT-100, Coulbourn Instruments). Tone-control rats were exposed to the tones but did not receive a shock.

### Day 17 - Fear extinction

Three days after the end of CUS, all rats received a single fear extinction session in Context B. All rats were exposed to 16 tone-alone trials with no shock, with an average inter-trial interval of 120 s.

### Chronic Unpredictable stress (CUS)

Chronic unpredictable stress was administered as previously described [17, 28]. For males, CUS consisted of 14 days of varied acute stressors: 30-min restraint, 1 h shake, 45 min social defeat, 10 min tail pinch, 24 h wet bedding, and 15 min mild footshock. We have reported that 14 days of CUS is not sufficient to induce set shifting deficits in females, so 21-day CUS was used for females, which induces the same set-shifting deficit as 14-day CUS in males [28]. CUS for females entailed mild footshock, tail pinch, shake stress, restraint, 24 hr wet bedding, and overnight lights on in place of social defeat.

### Attentional set shifting test (AST)

The AST was used to measure cognitive flexibility on the extra-dimensional set shifting task as previously described [29]. A week prior to testing, animals were food restricted to 12 g/day for males and 9 g/day for females (~66% of average daily intake). Animals were trained and tested in a white plastic arena containing a start gate at one end and two terracotta pots separated by a Plexiglas wall at the other end. The AST consists of three days (habituation, training, and testing), with an extra day inserted between training and testing to allow for the administration of extinction:

i.Habituation day: Rats were trained to dig in pots filled with sawdust to retrieve a food reward, half a Honey Nut Cheerio (General Mills Cereals, Minneapolis, MN, USA).

ii. Training day: Rats were trained to locate the reward in one of two pots by discriminating cues in two stimulus dimensions: the odor applied to the rim of the pot or the texture of the digging medium that filled the pot.

iii. Treatment day: Rats received bilateral microinjections and extinction or tone control treatment.

iv. Testing day: The day after extinction, rats were tested on a series of discrimination tasks, in which a criterion of six correct consecutive trials was required to proceed to the next task (Table 1). Rats were tested using either medium or odor as the relevant dimension in the early discrimination tasks (i.e., simple discrimination, complex discrimination, reversal learning, intradimensional shift, second reversal), leading to the formation of a cognitive set. In the extra-dimensional (ED) set shifting task, the previously relevant dimension (e.g., odor), was irrelevant while the previously irrelevant dimension (medium) now indicated the location of the reward.

**Table 1:** Stages of the Attentional Set Shifting Test (AST). Order of discrimination stages used for AST. All data shown are from the extra dimensional (ED) set shifting task.

	Dimensions		Example combinations	
Discrimination stage	Relevant	Irrelevant	(+)	(−)
Simple	Odor		Clove/sawdust	Nutmeg/sawdust
Compound	Odor	Medium	Clove/raffia	Nutmeg/yarn
			Clove/yarn	Nutmeg/raffia
Reversal 1	Odor	Medium	Nutmeg/raffia	Clove/yarn
			Nutmeg/yarn	Clove/raffia
Intradimensional shift	Odor	Medium	Rosemary/wood balls	Cinnamon/plastic beads
			Rosemary/plastic beads	Cinnamon/wood balls
Reversal 2	Odor	Medium	Cinnamon/plastic beads	Rosemary/wood balls
			Cinnamon/ wood balls	Rosemary/plastic beads
Extradimensional shift (ED)	Medium	Odor	Velvet/citronella	Crepe/thyme
			Velvet/thyme	Crepe/citronella

### Western blotting

17 days after rats were fear conditioned or received tone control treatment, rats underwent fear extinction and 30 min later were sacrificed via rapid decapitation. In experiments where animals were stressed, rats were fear conditioned on day 0, then received stress or handling from days 1–14 for males or 1–21 days for females. On days 15-16 (days 22-23 for females), rats were left undisturbed to coincide with the experimental timeline of the AST experiments. On day 17 (24 for females), rats received fear extinction and were sacrificed 30 min after the end of extinction. The IL was dissected on ice from a 2 mm coronal slab cut 2–4 mm caudal to the frontal pole. Cortex adjacent and medial to the internal capsule was dissected from this slab, frozen in isopentane on dry ice, and stored at −80 °C until use. In preliminary experiments measuring phosphorylation of Y515 in IL at 0’, 30’, and 60’ post extinction, we found an increase in phosphorylation of Y515 at 30 min post extinction. Thus, we selected 30’ as our tissue collection time. Western blots were performed as described previously [30]. Membranes were incubated in anti-rabbit or mouse secondary antibody (1:5,000 Cell Signaling) detected using ECL Prime (GE Healthcare, Little Chalfont, UK). Membranes were stripped and re-probed with antibodies against total TrkB (1:1000, Neuromics, [31]), ERK (rabbit pAb 1:5000, Santa Cruz Biotechnology, sc-94), or Akt (mouse mAb 1:1,000, CST 29202). Images were captured using the G:BOX-XT4 Chemi system (Syngene; Frederick,MD). All experimental group samples were normalized to the mean of tone control samples run in the same assay.

### Extinction with microinjections of BDNF neutralizing antibody and signaling pathway inhibitors

Rats were implanted with microinjection guide cannulae (Plastics One, Roanoke, VA) positioned 1 mm above the IL (AP + 2.9, ML −3.1, DV −3.8) at a 30° angle to minimize disruption of the prelimbic cortex. After the completion of experiments, a subset of rat brains from each group was used to verify cannula placement. Animals recovered for 1 week after surgery. On day 0, they received fear conditioning or tone exposure and on days 1–14 (1–21 for females), they underwent CUS. In this experiment and the next, in which exogenous BDNF was administered (below), males received overnight lights on in place of social defeat to make their CUS protocol identical to that of females except for the duration. There was no difference in the effect of CUS. On days 15–16, they were habituated and trained on the AST. On day 17, they received bilateral microinjection prior to extinction training. Infusion cannulae were inserted, extending 1 mm beyond the guide cannulae. Rats received bilateral injections of sheep IgG or sheep anti-BDNF (EMD Millipore, Billerica, MA), 0.5 µg/0.5 µl per side at a rate of 0.1 μl/min prior to extinction. This dose was shown to block BDNF signaling [24]. The injector remained in place for 2 min for diffusion. Twenty minutes after completing microinjections, rats underwent extinction. 24 h following extinction and microinjections, rats were tested on the set shifting test. For experiments involving kinase inhibitors, rats received bilateral microinjections of the PI3k inhibitor LY294002 (4.3 ng/0.5 μl/side), the Erk inhibitor PD98059 (2 µg/0.5 µl/side), or vehicle. As the different vehicles (0.5% DMSO in saline, 70% DMSO in saline, or saline alone) did not differ and had no effect on behavior, these groups were combined into a single vehicle group to minimize animal usage. Both LY294002 and PD98059 at the selected doses effectively inhibit phosphorylation of Akt and Erk in vivo, respectively [30, 32]. Based on preliminary western blot experiments showing phosphorylation of TrkB at Y515 occurred 30 mins post FE, we administered the inhibitors immediately after the end of the 32-minute extinction session so drug administration would precede induction of phosphorylation of Y515. One day later, rats were tested on the AST.

### Microinjections of BDNF peptide and signaling pathway inhibitors in place of extinction

Rats were implanted with microinjection guide cannulae (Plastics One, Roanoke, VA) positioned 1 mm above the IL (AP + 2.9, ML −3.1, DV −3.8) at a 30° angle. Animals recovered for one week after surgery. On days 1–14 for males and days 1–21 for females, rats underwent CUS. On days 15-16 (22–23 for females) they were habituated and trained on the AST. On day 17 (24 for females), they received bilateral microinjections (0.75 µl/side) into IL of BDNF alone (0.5 µg/µl, R&D Systems), or a combination of BDNF after either the PI3k inhibitor LY294002 (4.3 ng/0.5 µl/side), the Erk inhibitor PD98059 (2 µg/0.5 µl/side), or vehicle (0.5% DMSO in 0.9% saline or saline). The inhibitors were injected first, followed by BDNF. The microinjector remained in place for 2 min to allow diffusion. 24 h after microinjections, rats were tested on the set shifting test.

## Results

### The BDNF receptor TrkB is phosphorylated in IL at Y515 but not Y816 after fear extinction

**Fig. 1:**
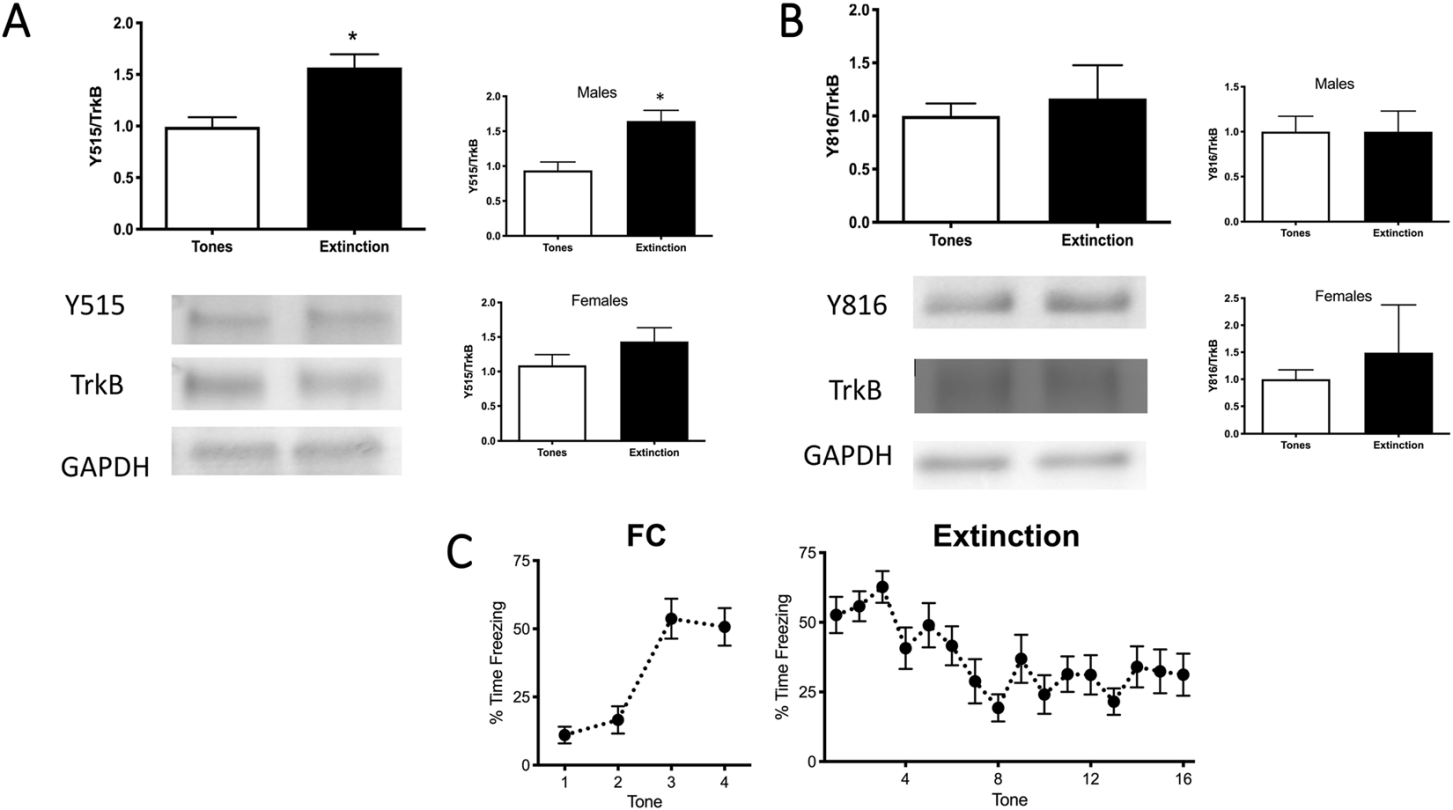
Extinction induces the phosphorylation of the BDNF receptor TrkB at Y515, but not Y816 in the IL. **A** Phosphorylation of TrkB at Y515 is increased in the IL 30 min after the end of extinction compared to tone controls (*p = 0.0018, *n* = 14–18/group). Insets show data for males and females separately; a student’s *t*-test showed an effect of extinction on Y515 in males (*t*_17 _= 3.568, *p* = 0.0024) but not in females alone. **B** Phosphorylation of TrkB at Y816 was unchanged 30 min after the end of extinction compared to tone controls (*p* = 0.6292, *n* = 12/group), insets show males and females separately. Bars represent SEM. **C** Fear conditioning (left) and extinction (right) curves showing % time freezing during the presentation of the tone. Bars represent SEM.

32 rats were used in 2 groups (tone controls vs. extinction), subsets of which were used to assess phosphorylation of TrkB at Y515 (9–10 males, 5–8 females), and Y816 (7–8 males, 4–5 females) in the IL cortex. In the aggregate analysis, extinction increased phosphorylation of TrkB at Y515 compared to tone controls in the IL 30 min after extinction (*t*_30_ = 3.432, *p* = 0.0018, Fig. 1A). Broken out by sex, a student’s *t*-test showed an effect of extinction on Y515 in males (*t*_17_ = 3.568, *p* = 0.0024). In females, the effect of extinction on Y515 phosphorylation was not significant, although these experiments were not specifically powered for separate analyses by sex (*t*_11_ = 1.222, *p* = 0.2474). Extinction did not induce an increase at Y816 (*t*_22_ = 0.4897, *p* = 0.6292; Fig. 1B). There was no effect of extinction on Y816 in either males (7-8/group; *t*_13_ = 0.00213, *p* = 0.9983, Fig. 1B, top inset) or females (4–5/group; *t*_7_ = 0.6168, *p* = 0.5569; Fig. 1B, bottom inset). Therefore, 30 min after extinction, BDNF-TrkB signaling is initiated in the IL by phosphorylation of Y515 but not Y816. Fig. 1C shows the typical pattern of freezing behavior during presentation of the tone during fear conditioning and extinction.

### The BDNF receptor TrkB is phosphorylated at Y515 in the IL of both stressed and control animals after the extinction

**Fig. 2:**
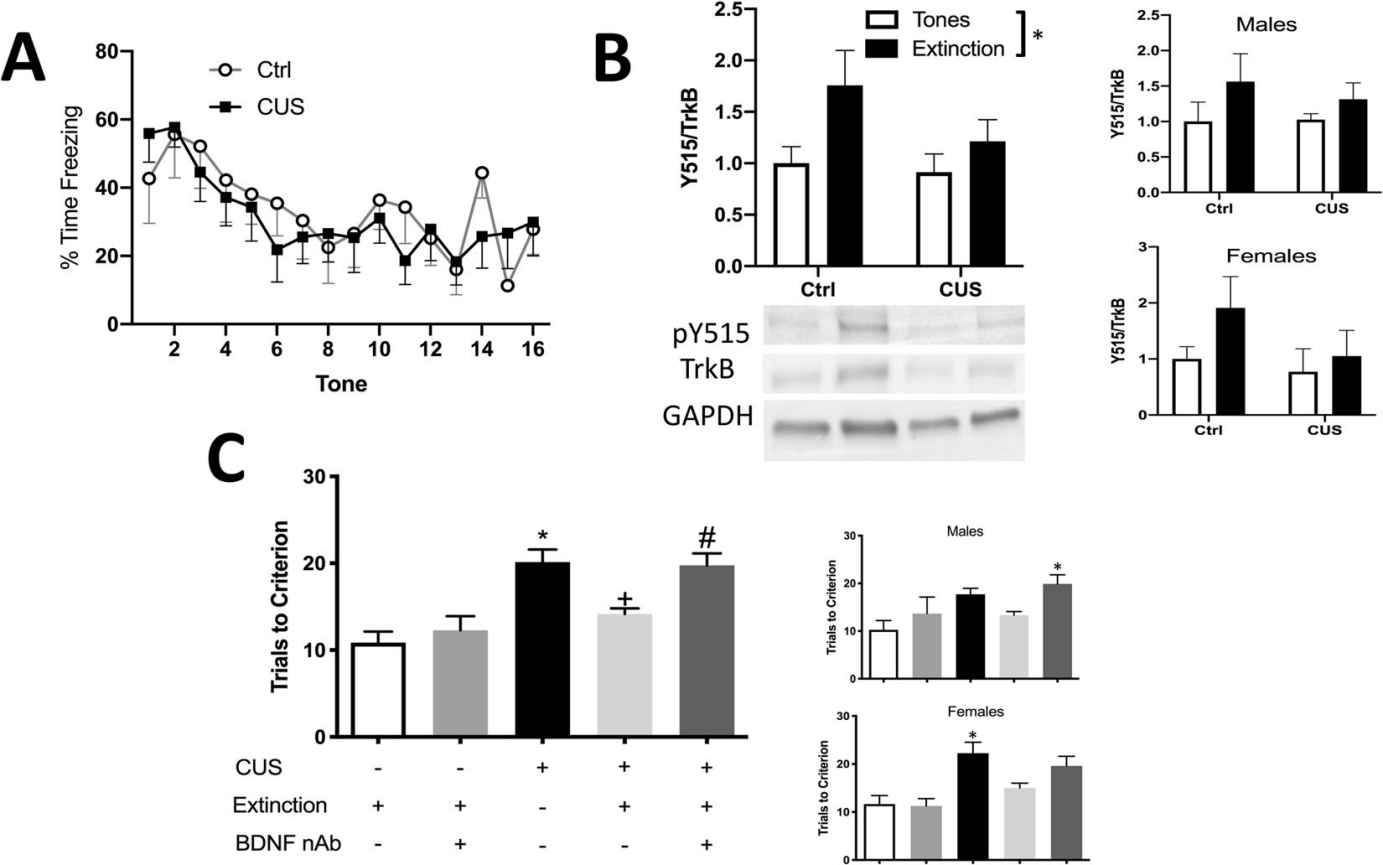
Infralimbic BDNF is necessary for the effects of extinction on set shifting in stressed animals. **A** Fear extinction curves in stressed and non-stressed rats, showing percent time freezing during tone presentation. Bars represent SEM. **B** Extinction induced an increase in phosphorylation of TrkB at Y515 compared to tone controls (*p = 0.0315; 8–9 rats/group). Insets show males and females separately. **C** CUS impaired set shifting on the AST (CUS/Tones/IgG compared to No Stress/Extinction/IgG, *p = 0.0003), and extinction rescued set shifting in stressed rats (CUS/Extinction/IgG compared to CUS/Tones/IgG, +*p* = 0.0091). Blocking BDNF in the IL at the time of extinction attenuated the beneficial effect of extinction in stressed rats (CUS/Extinction/anti-BDNF compared to CUS/Extinction/IgG, #*p* = 0.0218). The BDNF antibody alone had no effect in non-stressed control rats (No stress/Extinction/anti-BDNF compared to No stress/Extinction/IgG, *p* = 0.9739). Insets show males and females separately. In males, multiple comparisons showed a difference between No stress/extinction/control IgG vs CUS/extinction/anti-BDNF. In females, multiple comparisons showed differences between No stress/extinction/control IgG vs CUS/tones/IgG. Bars represent SEM.

To test whether extinction also increases phosphorylation at Y515 in stressed rats, 35 rats (18 males, 4–9/group; 17 females, 4–5/group) were used in 4 groups (stress or unstressed x extinction or tone controls) for western blotting. There was no difference in extinction between stress and control groups (*F*_1,16_ = 0.03366, *p* = 0.8567, Fig. 2A). ANOVA revealed a main effect of extinction, indicating an increase in phosphorylation of Y515 in IL (*F*_1,31 _= 5.073, *p* = 0.0315). There was no interaction (*F*_1,31 _= 0.9371, *p* = 0.3405), and no main effect of stress (*F*_1,31 _= 1.794, *p* = 0.1901; Fig. 2B). When data were analyzed separately by sex (Fig. 2B insets), the results were similar but did not reach significance (for males: stress, *F*_1__,__14 _= 0.2007, *p* = 0.6610; extinction, *F*_1__,__14 _= 2.844, *p* = 0.1138; interaction, *F*_1__,__14 _= 0.3002, *p* = 0.5924; for females: stress, *F*_1,13 _= 1.794, *p* = 0.2034; extinction (*F*_1,13 _= 1.499, *p* = 0.2425; interaction, *F*_1,13 _= 0.5060, *p* = 0.4894). There was no correlation between extinction, expressed as the freezing index (mean freezing at plateau on tones 9–16 / mean initial freezing on tones 2–4) and pTrkB Y515 levels (*r*_18_ = −0.2789, *p* = 0.2623).

### BDNF activity in IL cortex during extinction is necessary for its effects on set shifting

In total, 54 rats (28 males, 3–7/group; 26 females, 3–8/group) were used in five groups: (1) control group with control IgG in the IL (No stress/extinction/control IgG), (2) control group with neutralizing antibody against BDNF in the IL (No stress/extinction/anti-BDNF), (3) stress + tone control (CUS/tones/IgG), (4) extinction treatment (CUS/extinction/IgG), and 5) a group to test the necessity of BDNF for the effects of extinction (CUS/extinction/anti-BDNF). ANOVA revealed a significant group effect on set shifting (*F*_4,49 _= 9.544, *p* < 0.0001; Fig. 2C). Pairwise comparisons using the Holm–Sidak test showed that CUS impaired set shifting (CUS/Tones/IgG compared to No Stress/Extinction/IgG, *p* = 0.0003). Extinction rescued set shifting in stressed rats (CUS/Extinction/IgG compared to CUS/Tones/IgG, *p* = 0.0091), and blocking BDNF in the IL attenuated the beneficial effect of extinction (CUS/Extinction/anti-BDNF compared to CUS/Extinction/IgG, *p* = 0.0218). There was no effect of BDNF antibody alone on set shifting in non-stressed rats (No stress/Extinction/anti-BDNF compared to No stress/Extinction/IgG, *p* = 0.9739). These results suggest that BDNF activity in IL cortex is necessary during extinction for its beneficial effect on set shifting in stressed animals. As reported previously [17, 29] there was no effect of the treatments on fear extinction, as the extinction curves did not differ between groups (*F*_5,47 _= 0.8359, *p* = 0.5310). Analysis of male data alone revealed a significant group effect on set shifting (*F*_4,23 _= 4.662, *p* = 0.0067). Holm–Sidak multiple comparisons showed a difference between No stress/extinction/control IgG vs CUS/extinction/anti-BDNF (*p* = 0.0110); other group comparisons were not significant. ANOVA also revealed a significant group effect in females (*F*_4,21 _= 5.726, *p* = 0.0028); Holm–Sidak multiple comparisons showed differences between No stress/extinction/control IgG vs CUS/tones/IgG (*p* = 0.0263).

### Extinction induces the phosphorylation of Erk but not Akt in the IL of stressed and control animals

**Fig. 3:**
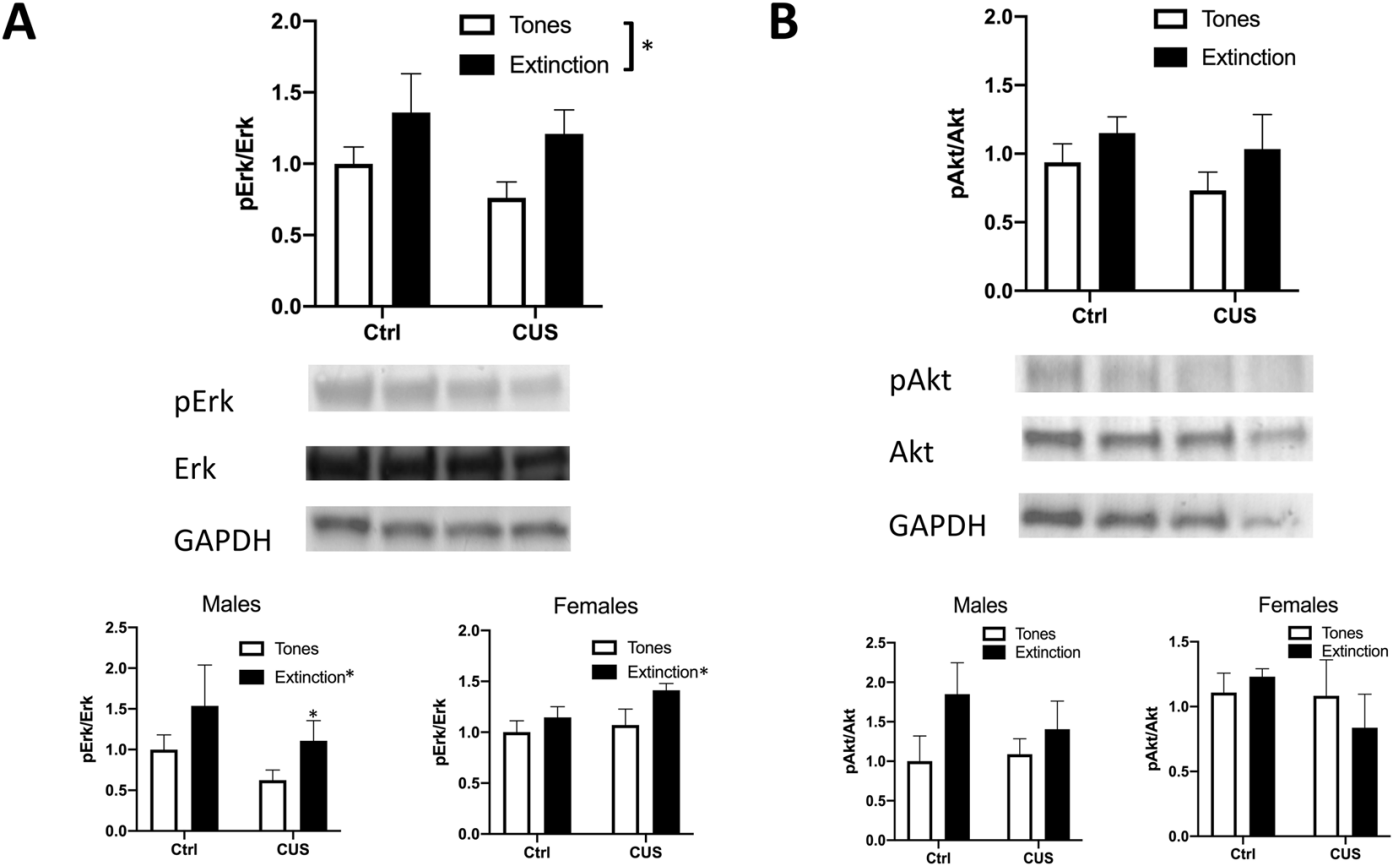
Extinction induces the phosphorylation of Erk but not Akt in the IL. **A** Extinction increased phosphorylation of Erk in the IL 30 min after extinction compared to tone controls (*p = 0.0021, *n* = 12–14/group). Insets show males and females separately. ANOVA on male data showed a main effect of extinction on pErk (*F*_1,30 _= 8.469, *p* = 0.0067), a main effect of stress (*F*_1,30 _= 5.136, *p* = 0.038), and a difference between Tones CUS vs Extinction Control (*p* = 0.0074). Extinction induced an increase in pErk in females and a difference between Tones-CUS vs Extinction-CUS *p* = 0.0023). **B** Extinction did not significantly increase the phosphorylation of Akt in the IL 30 min after the end of extinction compared to tone controls (*p* = 0.1639, *n* = 11–14/group). Insets show males and females separately.

In total, 51 rats (34 males, 8–10/group, 23 females, 4–7/group) were used in four groups (stress x extinction) to assess phosphorylation of Erk in the IL after extinction. ANOVA revealed a main effect of extinction (*F*_1,47 _= 10.60, *p* = 0.0021; Fig. 3A), no interaction (*F*_1,47 _= 0.4968, *p* = 0.4844), and no main effect of stress (*F*_1,47 _= 2.375, *p* = 0.1300). ANOVA on male data showed a main effect of extinction on pErk (*F*_1,30 _= 8.469, *p* = 0.0067), a main effect of stress (*F*_1,30 _= 5.136, *p* = 0.038), and no significant interaction (*F*_1,30 _= 0.1545, *p* = 0.6971). Holm–Sidak multiple comparisons showed a difference between Tones CUS vs Extinction Control (*p* = 0.0074). Extinction induced an increase in pErk in females (*F*_1,19 _= 10.07, *p* = 0.005) and a significant interaction (*F*_1,19 _= 4.75, *p* = 0.0421), but no main effect of stress (*F*_1,19 _= 0.5888, *p* = 0.4523). Holm–Sidak multiple comparisons showed a difference between Tones-CUS vs Extinction-CUS in females (*p* = 0.0023).

54 rats (25 males, 5–8/group; 18 females, 5–7/group) were used to assess pAkt. ANOVA revealed no significant main effect of stress (F_1,45 _= 2.038, p = 0.1603), no effect of extinction (F_1,45_ = 0.8007, p = 0.3756), and no interaction (F_1,45_ = 0.05960, p = 0.8082; Fig. 3B). Also, no significant effects were seen when data were analyzed separately by sex.

### The PI3k-Akt and MAPK-Erk signaling pathways are involved in the therapeutic effects of extinction on set shifting after stress

**Fig. 4:**
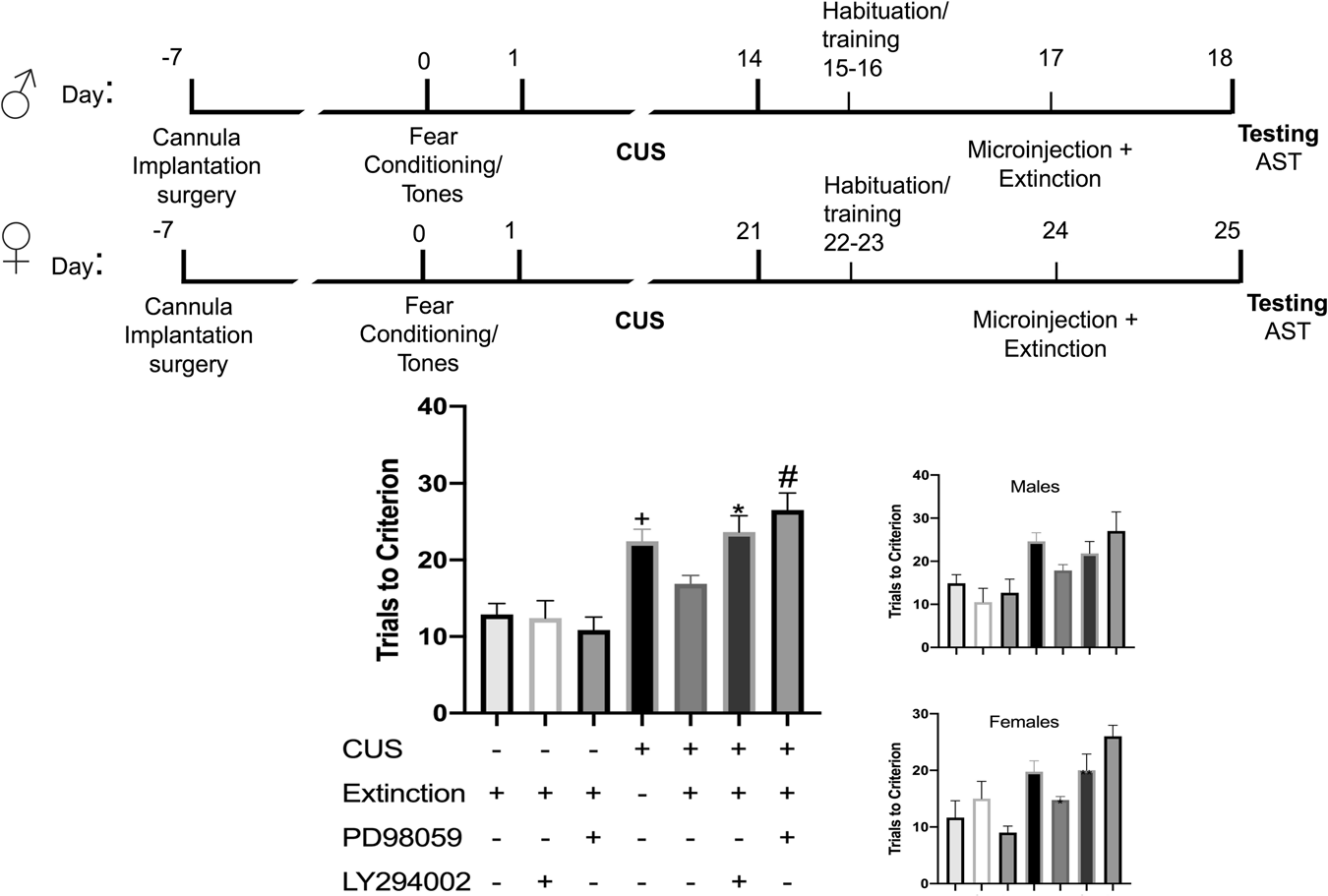
Erk and Akt signaling are required in the IL cortex for the beneficial effects of extinction on set shifting in stressed animals. Microinjections of either LY200492 or PD98059 into the IL cortex immediately after extinction blocked the effects of extinction on set-shifting 24 h later in stressed rats. CUS induced a deficit in set shifting (CUS Tones Veh vs Ctrl Ext Veh, +*p* = 0.0026). Both PD98059 and LY294002 attenuated the improvement in set-shifting induced by extinction in stressed animals (CUS Ext Veh vs CUS Ext PD98059: #*p* = 0.0031; CUS Ext Veh vs CUS Ext LY294002: *p = 0.0389). Insets show males and females separately.

In total, 62 rats (35 males, 3–7/group; 27 female rats, 3–5/group) were used in seven groups: (1) a control group (no stress/vehicle/extinction), (2) a control group with PI3k inhibitor LY294002 alone (no stress/LY294002/extinction), (3) a control group with MAPK inhibitor PD98059 alone (no stress/PD98059/extinction), (4) stress + tone control group (CUS/vehicle/tones), (5) extinction treatment (CUS/vehicle/extinction), (6) a group to determine the effect of blocking PI3k on the effects of extinction (CUS/LY294002/extinction), and (7) a group to determine the effects of blocking MAPK on the effects of extinction (CUS/PD98059/extinction). ANOVA revealed a significant group effect (*p* < 0.0001, *F*_6,55 _= 11.44; Fig. 4). Holm–Sidak post hoc tests showed an effect of stress (CUS Tones Veh vs Ctrl Ext Veh, *p* = 0.0026). Neither inhibitor alone affected set shifting in control animals (Ctrl Ext Veh vs Ctrl Ext LY294002: *p* = 0.95, Ctrl Ext Veh vs Ctrl Ext PD98059: *p* = 0.93). Extinction again had a beneficial effect after stress, as the performance of extinction-treated stressed animals was comparable to control animals (Ctrl Ext Veh vs CUS Ext Veh, *p* = 0.63). Both PD98059 and LY294002 attenuated performance in stressed animals that received extinction (CUS Ext Veh vs CUS Ext PD98059: *p* = 0.0031; CUS Ext Veh vs CUS Ext LY294002: *p* = 0.0389). Therefore, blocking either MAPK/Erk or PI3k/Akt signaling immediately after extinction blocked the beneficial effects on set shifting tested 24 h after extinction in stressed animals. As above, there was no effect of the treatments on extinction itself, as the extinction curves did not differ between groups (*F*_3,34 _= 0.5311, *p* = 0.6640). One way ANOVA on male data only revealed a significant group effect (*F*_6,27 _= 5.3879, *p* = 0.0009, Fig. 4 inset). However, as this experiment was not specifically powered to analyze separately by sex, Holm–Sidak multiple comparisons revealed no significant differences between groups: CUS Ext Veh vs CUS Ext PD98059 (*p* = 0.2896), Ctrl Ext Veh vs CUS Tones Veh (*p* = 0.0555), CUS Ext Veh vs CUS Ext PD98059 (*p* = 0.2896), CUS Ext Veh vs CUS Ext LY294002 (*p* = 0.7769). One way ANOVA on female data also revealed a significant group effect (*F*_6,19 _= 6.937981, *p* = 0.0005), but Holm–Sidak multiple comparisons revealed no specific group differences: Ctrl Ext Veh vs CUS tones Veh (*p* = 0.4167), CUS Ext Veh vs CUS Tones Veh (*p* = 0.7460), CUS Ext Veh vs CUS Ext LY294002 (*p* = 0.0546) and CUS Ext Veh vs CUS Ext PD98059 (*p* = 0.0662).

### BDNF alone reverses the effects of CUS on set shifting, and Erk signaling but not PI3k signaling is necessary for the effects of BDNF

**Fig. 5:**
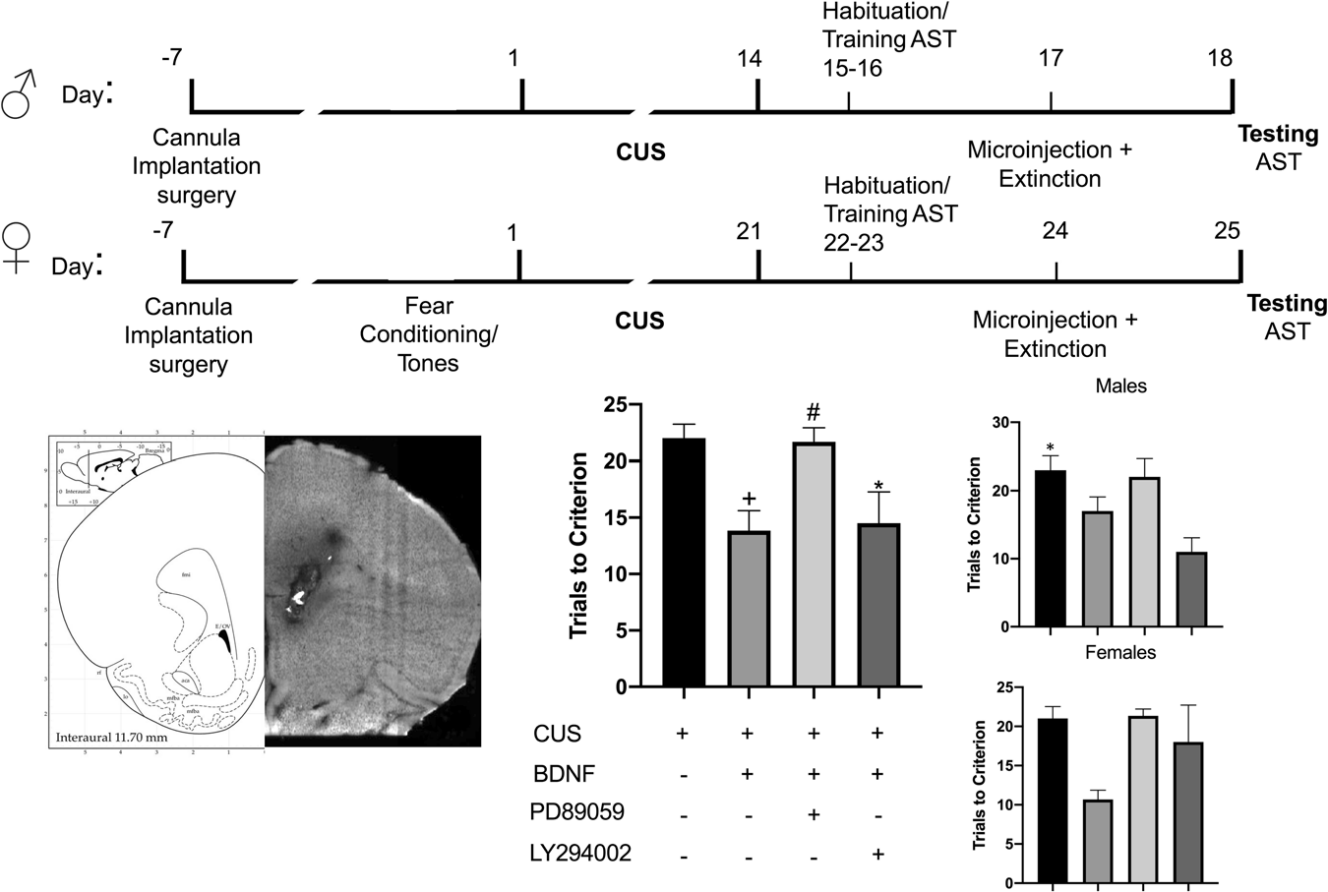
Exogenous BDNF administered in the IL cortex reverses the effects of stress on set shifting, and these effects are dependent on Erk signaling. CUS-BDNF animals performed better than CUS-vehicle-treated animals (+*p* = 0.0349, *n* = 6 rats/group). Stressed rats that received BDNF and the PI3k inhibitor LY294002 performed better than CUS-Veh animals (*p = 0.0408), and were not significantly different than CUS-BDNF alone (*p* = 0.9616). By contrast, stressed rats treated with BDNF and the Erk inhibitor PD90859 performed worse than CUS-BDNF rats (#*p* = 0.0385). Insets show males and females separately. In males, Holm–Sidak tests showed a difference between CUS-Veh and CUS/BDNF/LY294002 (*p* = 0.0312). Bars represent SEM. Bottom left is a representative example of injection site localization by dye injection into the IL.

In total, 24 rats (12 males, 3/group; 12 females, 3/group) were used in four groups: (1) a stress group (CUS/saline), (2) a group to determine whether BDNF alone microinjected into IL cortex in place of extinction is sufficient to reverse the effects of stress (CUS/BDNF), (3) a group to test whether the effects of BDNF are blocked by the PI3k inhibitor (CUS/BDNF/LY294002), and (4) a group to test whether the effects of BDNF are blocked by the MAPK inhibitor (CUS/BDNF/PD98059). ANOVA revealed a significant group effect (*F*_3,20 _= 5.602, *p* = 0.0059, Fig. 5). Multiple comparisons Holm–Sidak test showed that CUS-BDNF animals performed better than CUS-vehicle animals (*p* = 0.0349), while CUS-BDNF-PD90859 treated rats did not perform better than CUS-Veh animals (*p* = 0.9616). By contrast, stressed rats that received BDNF and the PI3k inhibitor still performed better than CUS-Veh animals (*p* = 0.0408), and were not significantly different than CUS-BDNF alone (p = 0.9616). Further, rats treated with BDNF and the Erk inhibitor PD90859 performed worse than CUS-BDNF (*p* = 0.0385). Therefore, BDNF alone in the IL cortex reversed the effects of stress on set shifting, and this effect was blocked by inhibiting Erk signaling, but not PI3k. One way ANOVA on male data alone revealed a significant group effect *F*_3,8 _= 6.050, *p* = 0.0187, Fig. 5). Holm–Sidak revealed no differences between CUS–Veh males compared to CUS-BDNF males (*p* = 0.3273), but did detect a difference between CUS-Veh and CUS/BDNF/LY294002 (*p* = 0.0312). No differences were observed in CUS-BDNF vs CUS/BDNF/ PD98059 in males (*p* = 0.3273). A one way ANOVA revealed no group differences in female data analyzed separately (*F*_3,8 _= 3.652, *p* = 0.0635); thus, pairwise comparisons were not performed.

## Discussion

The results of this study demonstrate the necessity of BDNF activity in the IL for the beneficial effects of extinction on restoring stress-induced cognitive deficits in both male and female rats. Our findings are consistent with previous work highlighting the role of BDNF in extinction-mediated plasticity [21]. In humans, the BDNF Val66Met polymorphism, a single nucleotide polymorphism associated with reduced BDNF release, increases the risk of developing PTSD, and is associated with a poorer response to exposure therapy [33, 34]. Thus, compounds that enhance fear extinction by promoting BDNF activity may be clinically relevant to the treatment of PTSD and MDD [11]. Following exposure therapy, symptoms may take weeks or months to improve, and a small number of patients achieve remission. Our results suggest that pharmacotherapies enhancing BDNF/Erk signaling in the mPFC may be advantageous as adjuncts to behavioral therapy. For example, studies have demonstrated the role of BDNF and Erk signaling in the antidepressant effects of ketamine [35], which is effective in treatment-resistant patients. Indeed, a recent pilot study showed that extinction and reconsolidation combined with adjunct administration of ketamine were effective in treating PTSD [36].

Several studies have demonstrated a role for BDNF in restoring mPFC function compromised by stress [37, 38]. Chronic stress induces functional and morphological alterations in the mPFC, such as dendritic atrophy of pyramidal neurons and reduced expression of glutamate receptors [39, 40], whereas BDNF induces long-term potentiation (LTP), enhances neuronal excitability, glutamate receptor trafficking, and promotes local protein synthesis in dendrites [41]. Extinction restores mPFC responsivity compromised by stress [17], and requires de novo protein synthesis in the IL for its beneficial effects. The current results suggest that BDNF activity in the IL during extinction facilitates the restoration of mPFC responsivity after stress, perhaps by promoting the synthesis of plasticity-related proteins. This hypothesis requires further testing. We observed phosphorylation of the BDNF receptor TrkB in IL after extinction, consistent with reports suggesting the activity-dependent release of BDNF during extinction [21]. After extinction learning, BDNF is increased in ventral hippocampal neurons [42]. Release of BDNF from ventral hippocampal projections to the IL is crucial for extinction memory [42]. Local release of BDNF elicits dendritic growth [43], a mechanism through which extinction-induced BDNF release in IL may reverse the detrimental effects of stress.

Inducing plasticity in the IL via extinction may also reverse stress-induced behavioral impairments mediated in other brain regions innervated by IL cortex. During extinction, the IL exerts inhibitory control over the amygdala to suppress fear. In individuals with PTSD, mPFC hypoactivity and amygdala hyperresponsivity are predictive of poor response to cognitive behavioral therapy [44]. Restoring IL function via BDNF signaling may therefore correct amygdala hyperresponsiveness and exaggerated fear response by enhancing activity in the mPFC. The IL also projects to the lateral septum (LS), which mediates active coping behavior in the shock probe defensive burying test (SPDB) [45, 46]. We have reported that extinction corrects the maladaptive shift from active to passive coping in the SPDB after chronic stress, and this effect relies on IL activity [17]. Thus, BDNF-induced plasticity in IL cortex during extinction may improve active coping behavior by facilitating modulatory regulation of LS activity by IL after stress.

The current results also elucidate intracellular signaling mechanisms potentially involved in the effects of extinction. Both Akt and Erk signaling contributed to the beneficial effects of extinction on set shifting that had been compromised by stress. Both Akt and Erk signaling contribute to long-term memory formation [47, 48]. Therefore, inhibiting these pathways likely interferes with extinction memory consolidation in the IL, which may reduce the subsequent therapeutic effects of extinction after stress. Extinction induced a significant increase in Erk phosphorylation, but only a modest, non-significant increase in phosphorylated Akt, consistent with previous reports [22]. This may seem at odds with the demonstration that PI3k blockade attenuated the beneficial effects of extinction. However, it is possible that only a small fraction of the total pool of Akt is activated by extinction, or that an increase in Akt phosphorylation only occurs in some cells after extinction, making it difficult to detect against a large unaffected background. Our results suggest, however, that even if the increase in phosphorylation is modest after extinction, Akt signaling is nonetheless necessary for the beneficial effects of extinction on set shifting. Another possibility is that, in addition to BDNF activity, extinction may also invoke other processes and transmitters that require basal PI3k-Akt activity in the IL, independent of induction by BDNF, to promote plasticity beneficial to set shifting. For example, Insulin Growth Factor-2 signals via PI3k-Akt, and has been shown to be involved in the consolidation of fear extinction, making it another potential candidate for inducing plasticity through actions of PI3k-Akt [49].

Administration of exogenous BDNF into the IL in lieu of extinction reversed the effects of stress on set shifting, and this required Erk, but not Akt signaling. Although Erk and Akt share common targets, these pathways have different roles in some BDNF-related processes [47–49]. These results are in agreement with studies demonstrating the importance of Erk signaling for memory- and BDNF-induced plasticity [50–52]. Erk activation is necessary for BDNF-induced dendritic spine formation [50], and extinction increases dendritic spine density after acute stress [53]. Moreover, BDNF requires Erk signaling to phosphorylate cyclic AMP response element-binding protein (CREB) which mediates memory phase transitions [54]. BDNF/Erk-induced CREB activation drives the expression of genes regulating LTP and neuronal plasticity, e.g., activity-regulated cytoskeleton-associated protein (Arc) and Zif268 [55]. Thus, it is likely that extinction, via BDNF-Erk signaling, induces structural and functional plasticity in the IL necessary for memory consolidation, and for the beneficial effects of extinction on stress-compromised cognitive function such as set shifting.

In sum, the current results replicated our previous reports that extinction reversed stress-induced deficits in set shifting [17, 29] in both males and females, and demonstrated that these beneficial effects of extinction are dependent on BDNF activity in the IL during extinction. Extinction phosphorylated TrkB at the Y515 residue, but not Y816, suggesting a potential role for Y515-initiated intracellular signaling for the effects of extinction. Inhibiting either Akt or Erk in the IL after extinction prevented the therapeutic effects of extinction in stressed animals, and extinction increased phosphorylation of Erk, but not Akt, in the IL cortex. We further showed that exogenous BDNF microinjected into the IL was sufficient to reverse the detrimental effects of stress on set shifting, mimicking the beneficial effects of extinction. The beneficial effects of BDNF alone were mediated by Erk, but not Akt signaling. This information may be useful in developing strategies for adjunctive treatment during exposure therapy for PTSD to enhance or accelerate therapeutic efficacy and improve treatment outcomes.

## References

[1] Moussavi S, Chatterji S, Verdes E, Tandon A, Patel V, Ustun B (2007). Depression, chronic diseases, and decrements in health: results from the World Health Surveys. Lancet.

[2] Rytwinski NK, Scur MD, Feeny NC, Youngstrom EA (2013). The co‐occurrence of major depressive disorder among individuals with posttraumatic stress disorder: a meta‐analysis. J Trauma Stress.

[3] Wiles N, Thomas L, Abel A, Ridgway N, Turner N, Campbell J (2013). Cognitive behavioural therapy as an adjunct to pharmacotherapy for primary care based patients with treatment resistant depression: results of the CoBalT randomised controlled trial. Lancet.

[4] de Kleine RA, Rothbaum BO, Van, Minnen A (2013). Pharmacological enhancement of exposure-based treatment in PTSD: a qualitative review. Eur J Psychotraumatology.

[5] Ben-Zion Z, Fine NB, Keynan NJ, Admon R, Green N, Halevi M (2018). Cognitive flexibility predicts PTSD symptoms: observational and interventional studies. Front Psychiatry.

[6] Peters AT, Jacobs RH, Crane NA, Ryan KA, Weisenbach SL, Ajilore O (2017). Domain‐specific impairment in cognitive control among remitted youth with a history of major depression. Early intervention psychiatry.

[7] Birrell JM, Brown VJ (2000). Medial frontal cortex mediates perceptual attentional set shifting in the rat. J Neurosci.

[8] Radley J, Sisti H, Hao J, Rocher AB, McCall T, Hof P (2004). Chronic behavioral stress induces apical dendritic reorganization in pyramidal neurons of the medial prefrontal cortex. Neuroscience.

[9] Beck AT. Cognitive therapy and the emotional disorders: Penguin; 1979.

[10] Walter KH, Palmieri PA, Gunstad J (2010). More than symptom reduction: changes in executive function over the course of PTSD treatment. J Trauma Stress: Off Publ Int Soc Trauma Stress Stud.

[11] Andero R, Ressler KJ (2012). Fear extinction and BDNF: translating animal models of PTSD to the clinic. Genes, Brain Behav.

[12] Foa EB, Rothbaum BO (1989). Behavioural psychotherapy for post-traumatic stress disorder. Int Rev Psychiatry.

[13] Milad MR, Quirk GJ (2012). Fear extinction as a model for translational neuroscience: ten years of progress. Annu Rev Psychol.

[14] Paredes D, Morilak DA (2019). A rodent model of exposure therapy: the use of fear extinction as a therapeutic intervention for PTSD. Front Behav Neurosci.

[15] Hassien AM, Shue F, Bernier BE, Drew MR (2020). A mouse model of stress-enhanced fear learning demonstrates extinction-sensitive and extinction-resistant effects of footshock stress. Behavioural Brain Res.

[16] Sierra-Mercado D, Padilla-Coreano N, Quirk GJ (2011). Dissociable roles of prelimbic and infralimbic cortices, ventral hippocampus, and basolateral amygdala in the expression and extinction of conditioned fear. Neuropsychopharmacology.

[17] Fucich EA, Paredes D, Saunders MO, Morilak DA (2018). Activity in the ventral medial prefrontal cortex is necessary for the therapeutic effects of extinction in rats. J Neurosci.

[18] King AP, Block SR, Sripada RK, Rauch SA, Porter KE, Favorite TK (2016). A pilot study of mindfulness-based exposure therapy in OEF/OIF combat veterans with PTSD: altered medial frontal cortex and amygdala responses in social–emotional processing. Front Psychiatry.

[19] Soliman F, Glatt CE, Bath KG, Levita L, Jones RM, Pattwell SS (2010). A genetic variant BDNF polymorphism alters extinction learning in both mouse and human. Science.

[20] Choi DC, Maguschak KA, Ye K, Jang S-W, Myers KM, Ressler KJ (2010). Prelimbic cortical BDNF is required for memory of learned fear but not extinction or innate fear. Proc Natl Acad Sci.

[21] Peters J, Dieppa-Perea LM, Melendez LM, Quirk GJ (2010). Induction of fear extinction with hippocampal-infralimbic BDNF. Science.

[22] Slouzkey I, Maroun M (2016). PI3-kinase cascade has a differential role in acquisition and extinction of conditioned fear memory in juvenile and adult rats. Learn Mem.

[23] Hugues S, Chessel A, Lena I, Marsault R, Garcia R (2006). Prefrontal infusion of PD098059 immediately after fear extinction training blocks extinction‐associated prefrontal synaptic plasticity and decreases prefrontal ERK2 phosphorylation. Synapse.

[24] Alonso M, Vianna MR, Izquierdo I, Medina JH (2002). Signaling mechanisms mediating BDNF modulation of memory formation in vivo in the hippocampus. Cell Mol Neurobiol.

[25] Duman RS, Voleti B (2012). Signaling pathways underlying the pathophysiology and treatment of depression: novel mechanisms for rapid-acting agents. Trends Neurosci.

[26] Barfield ET, Gerber KJ, Zimmermann KS, Ressler KJ, Parsons RG, Gourley SL (2017). Regulation of actions and habits by ventral hippocampal trkB and adolescent corticosteroid exposure. PLoS Biol.

[27] Infralimbic BDNF is necessary for the therapeutic effects of extinction after chronic stress in male and female rats [database on the Internet]. Society for Neuroscience. 2019.

[28] Bulin SE, Hohl KM, Paredes D, Silva JD, Morilak DA (2020). Bidirectional optogenetically-induced plasticity of evoked responses in the rat medial prefrontal cortex can impair or enhance cognitive set-shifting. Eneuro.

[29] Fucich EA, Paredes D, Morilak DA (2016). Therapeutic effects of extinction learning as a model of exposure therapy in rats. Neuropsychopharmacology.

[30] Girotti M, Silva JD, George CM, Morilak DA (2019). Ciliary neurotrophic factor signaling in the rat orbitofrontal cortex ameliorates stress-induced deficits in reversal learning. Neuropharmacology.

[31] Carreño FR, Walch JD, Dutta M, Nedungadi TP, Cunningham JT (2011). BDNF-TrkB pathway mediates NMDA receptor NR2B subunit phosphorylation in the supraoptic nuclei following progressive dehydration. J Neuroendocrinol.

[32] Kim S, Shin J-K, Yoon HS, Kim J-H (2011). Blockade of ERK phosphorylation in the nucleus accumbens inhibits the expression of cocaine-induced behavioral sensitization in rats. Korean J Physiol Pharmacol.

[33] Felmingham KL, Dobson-Stone C, Schofield PR, Quirk GJ, Bryant RA (2013). The brain-derived neurotrophic factor Val66Met polymorphism predicts response to exposure therapy in posttraumatic stress disorder. Biol Psychiatry.

[34] Zhang L, Benedek D, Fullerton C, Forsten R, Naifeh J, Li X (2014). PTSD risk is associated with BDNF Val66Met and BDNF overexpression. Mol Psychiatry.

[35] Duman RS, Li N, Liu R-J, Duric V, Aghajanian G (2012). Signaling pathways underlying the rapid antidepressant actions of ketamine. Neuropharmacology.

[36] Pradhan B, Mitrev L, Moaddell R, Wainer IW (2018). d-Serine is a potential biomarker for clinical response in treatment of post-traumatic stress disorder using (R, S)-ketamine infusion and TIMBER psychotherapy: a pilot study. Biochimica et Biophysica Acta (BBA)-Proteins Proteom.

[37] Xu H, Wang J, Zhang K, Zhao M, Ellenbroek B, Shao F (2018). Effects of adolescent social stress and antidepressant treatment on cognitive inflexibility and Bdnf epigenetic modifications in the mPFC of adult mice. Psychoneuroendocrinology.

[38] Tornese P, Sala N, Bonini D, Bonifacino T, La Via L, Milanese M (2019). Chronic mild stress induces anhedonic behavior and changes in glutamate release, BDNF trafficking and dendrite morphology only in stress vulnerable rats. The rapid restorative action of ketamine. Neurobiol Stress.

[39] Liston C, Miller MM, Goldwater DS, Radley JJ, Rocher AB, Hof PR (2006). Stress-induced alterations in prefrontal cortical dendritic morphology predict selective impairments in perceptual attentional set-shifting. J Neurosci.

[40] Jett JD, Bulin SE, Hatherall LC, McCartney CM, Morilak DA (2017). Deficits in cognitive flexibility induced by chronic unpredictable stress are associated with impaired glutamate neurotransmission in the rat medial prefrontal cortex. Neuroscience.

[41] Leal G, Comprido D, Duarte CB (2014). BDNF-induced local protein synthesis and synaptic plasticity. Neuropharmacology.

[42] Rosas-Vidal LE, Do-Monte FH, Sotres-Bayon F, Quirk GJ (2014). Hippocampal–prefrontal BDNF and memory for fear extinction. Neuropsychopharmacology.

[43] Horch HW, Katz LC (2002). BDNF release from single cells elicits local dendritic growth in nearby neurons. Nat Neurosci.

[44] Patel R, Spreng RN, Shin LM, Girard TA (2012). Neurocircuitry models of posttraumatic stress disorder and beyond: a meta-analysis of functional neuroimaging studies. Neurosci Biobehav Rev.

[45] Vertes RP (2004). Differential projections of the infralimbic and prelimbic cortex in the rat. Synapse.

[46] Treit D, Pesold C, Rotzinger S (1993). Dissociating the anti-fear effects of septal and amygdaloid lesions using two pharmacologically validated models of rat anxiety. Behav Neurosci.

[47] Slouzkey I, Rosenblum K, Maroun M (2013). Memory of conditioned taste aversion is erased by inhibition of PI3K in the insular cortex. Neuropsychopharmacology.

[48] Kelly Á, Laroche S, Davis S (2003). Activation of mitogen-activated protein kinase/extracellular signal-regulated kinase in hippocampal circuitry is required for consolidation and reconsolidation of recognition memory. J Neurosci.

[49] Agis‐Balboa RC, Arcos‐Diaz D, Wittnam J, Govindarajan N, Blom K, Burkhardt S (2011). A hippocampal insulin‐growth factor 2 pathway regulates the extinction of fear memories. EMBO J.

[50] Alonso M, Medina JH, Pozzo-Miller L (2004). ERK1/2 activation is necessary for BDNF to increase dendritic spine density in hippocampal CA1 pyramidal neurons. Learn Mem.

[51] Mullen LM, Pak KK, Chavez E, Kondo K, Brand Y, Ryan AFRas/p38 (2012). and PI3K/Akt but not Mek/Erk signaling mediate BDNF-induced neurite formation on neonatal cochlear spiral ganglion explants. Brain Res.

[52] Schafe GE, Atkins CM, Swank MW, Bauer EP, Sweatt JD, LeDoux JE (2000). Activation of ERK/MAP kinase in the amygdala is required for memory consolidation of pavlovian fear conditioning. J Neurosci.

[53] Moench KM, Maroun M, Kavushansky A, Wellman C (2016). Alterations in neuronal morphology in infralimbic cortex predict resistance to fear extinction following acute stress. Neurobiol Stress.

[54] Fukushima H, Zhang Y, Kida S (2021). Active transition of fear memory phase from reconsolidation to extinction through ERK-mediated prevention of reconsolidation.. J of Neurosci.

[55] Ying S-W, Futter M, Rosenblum K, Webber MJ, Hunt SP, Bliss TV (2002). Brain-derived neurotrophic factor induces long-term potentiation in intact adult hippocampus: requirement for ERK activation coupled to CREB and upregulation of Arc synthesis. J Neurosci.

